# A cross-sectional analysis of the vaginal microenvironment in rheumatoid arthritis

**DOI:** 10.1128/spectrum.03602-25

**Published:** 2026-04-30

**Authors:** Marlyd E. Mejia, Savannah Bowman, Jessica Lee, Ali El-Halwagi, Keshia Ferguson, Maryjo Maliekel, Yixuan Zhou, Camille Serchejian, Clare M. Robertson, Mallory B. Ballard, Lee B. Lu, Sobia Khan, Olubunmi O. Oladunjoye, Shixia Huang, Sandeep K. Agarwal, Kathryn A. Patras

**Affiliations:** 1 Department of Molecular Virology and Microbiology, Baylor College of Medicine, Houston, Texas, USA; 2 Section of Immunology, Allergy, and Rheumatology, Department of Medicine, Baylor College of Medicine, Houston, Texas, USA; 3 Section of General Internal Medicine, Department of Medicine, Baylor College of Medicine, Houston, Texas, USA; 4 Department of Molecular and Cellular Biology, Baylor College of Medicine, Houston, Texas, USA; 5 Alkek Center for Metagenomics and Microbiome Research, Baylor College of Medicine, Houston, Texas, USA; University of Nebraska-Lincoln, Lincoln, Nebraska, USA

**Keywords:** vaginal cytokines, rheumatoid arthritis, vaginal microbiome

## Abstract

The human microbiota is implicated in the development and progression of rheumatoid arthritis (RA). Given the increased RA burden in women and well-known correlations between the vaginal microbiota and local inflammation, we seek to understand the vaginal microenvironment in the context of RA pathology. Self-collected vaginal swabs and questionnaires on dietary, menstrual, and health information were obtained from 36 RA and 50 demographically-matched control women, 18–63 years of age. Medication regimen, along with disease activity and severity, was captured for the RA cohort. Vaginal swabs were subjected to long-read 16S rRNA gene sequencing, multiplex cytokine analyses, and quantification of rheumatoid factor, C-reactive protein, and anti-citrullinated protein antibodies (ACPAs). Vaginal microbial richness and *Peptoniphilus* and *Prevotella*, among other rare taxa, were elevated in RA versus control samples. Vaginal interleukin (IL)-18 and epidermeal growth factor (EGF) levels were increased in the RA group; IL-18 correlated with multiple microbial features, whereas EGF levels were not associated with bacterial composition or other host factors. When faceted by diet and menopausal status, several immune markers were increased in the RA vaginal environment. Vaginal ACPAs were higher in the RA group and positively correlated with *Streptococcus* and multiple vaginal inflammatory cytokines. We describe vaginal microbial and immunological differences in women with RA, particularly when accounting for diet and menopausal status, and disease activity and severity. This work opens a new avenue in the multidisciplinary approach to RA patient care.

**IMPORTANCE:**

Rheumatoid arthritis (RA) is a debilitating autoimmune disease that disproportionately impacts women. Although it is widely recognized that microbial factors can trigger or aggravate RA symptoms and alter disease progression, it is unknown whether RA impacts the microbiota and immune responses within the vaginal tract. In this study, we compare the vaginal microbial communities and immune (cytokine) profiles in women with RA and healthy controls. Within RA patients, we also evaluate how these factors relate to clinical RA symptoms, RA biomarkers, and RA-related medications. Overall, we found that RA was associated with increased microbial diversity and multiple inflammatory markers, some of which were also associated with RA biomarkers and disease activity. These findings suggest that the vaginal tract may be an additional tissue impacted by RA disease, and further research is needed to understand mechanisms and potential for therapeutic intervention.

## INTRODUCTION

Rheumatoid arthritis (RA) is an autoimmune disease affecting ~1% of the global population (1). RA manifests primarily as inflammatory arthritis characterized by synovial inflammation, leading to permanent joint damage and decreased quality of life. RA is diagnosed based on the presence of joint pain and synovitis, aided by the presence of anti-citrullinated protein antibodies (ACPA) and rheumatoid factor (RF). RA disease activity is followed using measures like the clinical disease activity index (CDAI), which incorporates patient and physician overall assessment of disease activity and number of swollen and tender joint counts. Severity and damage from RA is evaluated using radiographs to assess joint space narrowing and radiographic erosions (2). RA-related inflammation also has systemic implications and multi-organ involvement including elevated risk for cardiac disease, sleep disturbance, cognitive decline, and mucosal inflammation (3). These wide-reaching implications have highlighted the need for more integrated and multidisciplinary approaches to RA patient care.

Like other autoimmune diseases, RA is caused by a combination of underlying genetic factors and environmental triggers, including microbial perturbations (4–6). Experimentally, transfer of RA-associated gut microbes to arthritis-prone mice drives more severe synovial inflammation, suggesting a causal role for the microbiome in RA pathology (7, 8). Microbial molecular mimicry, bystander activation, epitope spreading, or production of cryptic antigens (i.e., post-translational citrullination via microbial enzymes) can trigger an immune response cascading into auto-reactivity (9). Particularly, the release of citrullinated oral bacteria into circulation during periodontal disease can activate ACPA-producing B cells and correlates with RA symptom flares (10). Unique oral and gastrointestinal microbial signatures, such as increased *Prevotella* sp., are observed in untreated, new-onset RA patients (11–13), indicating RA may influence microbial composition even in the absence of mucosal disease. These studies strongly implicate the microbiome in RA pathogenesis and may have important clinical implications for patients.

RA has a predilection to affect women (~70% of disease burden), with reproductive-age women having 3–5-fold increased RA incidence compared with male counterparts (4, 14). Notably, hormonal contraception or pregnancy relieves symptoms in ~75% of women, whereas disease aggravation is observed post-partum and post-menopause (15, 16). As with other mucosal sites, the vaginal microenvironment is impacted by environmental and host factors with particular sensitivity to hormonal fluctuations during menses, throughout pregnancy, and post-menopause (17, 18). A non-optimal vaginal microbiota, typically characterized by low levels of *Lactobacillus* species, is associated with local or systemic inflammation, increased susceptibility to infections, and adverse gynecologic or obstetric outcomes (17, 19, 20). Associations between RA and microbial constituents, and the importance of vaginal microbe-immune interactions in women’s health, warrant characterization of the vaginal microenvironment in RA.

Hypothesizing that the vaginal microbiota and mucosal immunity are dysregulated in RA, correlating with autoimmune responses and disease activity, we performed microbiome, cytokine, and autoimmune factor profiling in vaginal samples collected from women with and without RA. We further integrated medical history, demographic information, medication use, and RA disease activity and severity measures to resolve correlations within RA subgroups. We found that vaginal microbial and immunologic features are indicative of RA status and current disease activity, revealing the vaginal mucosa as a previously unrecognized site altered in RA.

## RESULTS

### Patient demographics and RA disease heterogeneity

Female RA patients (*n* = 36) and healthy controls (*n* = 50) were recruited for vaginal swab collection. Demographics including age, race and ethnicity, menopausal status, educational status, alcohol and cigarette use, sexual activity, and type 2 diabetes status were similar between groups whereas antibiotic use within the last 1–6 months was greater in the RA group (Table 1). Although diet categorization was not significantly different between cohorts, diet and subsequent BMI status influence both the vaginal microbiome and host inflammation. Shannon diversity of the vaginal microbiome is higher in women on a vegetarian diet (21), and obese women have a higher incidence of BV (22). The American College of Rheumatology further recommends that patients avoid red meat and food high in unsaturated fats (23). High BMI via a high-fat diet correlates with increased risk of RA, particularly in women, and inhibits amelioration of inflammation in a mouse model of RA (24, 25). Therefore, women with high fat and meat intake were binned into a “High” group, and those with low or no consumption of fats and meat were binned “Reduced.” Dietary breakdown, medical conditions including additional autoimmune diseases, current medications, and recent urogenital symptoms and infections are reported in Fig. S1. The RA cohort displayed heterogeneity in time since diagnosis (<1–22 years), CDAI, hematological and serological findings, and medication history (Fig. S2).

**TABLE 1:** Clinical demographics of study participants stratified by RA status

Characteristic	Overall *n* = 86^*a*^	Control *n* = 50^*a*^	Rheumatoid arthritis *n* = 36^*a*^	*P*-value^*b*^
Age	47 (21–62)	45 (23–62)	49 (21–62)	0.14
Clinic				0.001
Clinic 1	46 (53%)	34 (68%)	12 (33%)	
Clinic 2	40 (47%)	16 (32%)	24 (67%)	
Ethnicity				0.26
Asian	4 (4.7%)	3 (6.0%)	1 (2.8%)	
Black or African American	21 (24%)	14 (28%)	7 (19%)	
Hispanic/Latino	48 (56%)	23 (46%)	25 (69%)	
Native American or Alaskan Native	1 (1.2%)	1 (2.0%)	0 (0%)	
White	12 (14%)	9 (18%)	3 (8.3%)	
Menopausal status				0.49
Pre-menopause	35 (41%)	23 (46%)	12 (33%)	
Peri/menopause	25 (29%)	13 (26%)	12 (33%)	
Post-menopause	26 (30%)	14 (28%)	12 (33%)	
Diet (meat/fat intake)				0.063
High	57 (66%)	29 (58%)	28 (78%)	
Reduced	24 (28%)	16 (32%)	8 (22%)	
Not disclosed	5 (5.8%)	5 (10%)	0 (0%)	
Alcohol consumption				0.14
Once daily	2 (2.3%)	2 (4.0%)	0 (0%)	
Occasionally	20 (23%)	15 (30%)	5 (14%)	
Rarely	19 (22%)	11 (22%)	8 (22%)	
Not applicable	44 (51%)	21 (42%)	23 (64%)	
Not disclosed	1 (1.2%)	1 (2.0%)	0 (0%)	
Cigarette smoking				0.94
Multiple times a day	1 (1.2%)	0 (0%)	1 (2.8%)	
Occasionally	2 (2.3%)	1 (2.0%)	1 (2.8%)	
Rarely	5 (5.8%)	3 (6.0%)	2 (5.6%)	
Not applicable	77 (90%)	45 (90%)	32 (89%)	
Not disclosed	1 (1.2%)	1 (2.0%)	0 (0%)	
Education				0.16
Advanced degree	10 (12%)	5 (10%)	5 (14%)	
College/university degree	24 (28%)	19 (38%)	5 (14%)	
Some college	13 (15%)	7 (14%)	6 (17%)	
High school	23 (27%)	11 (22%)	12 (33%)	
Elementary/middle school	15 (17%)	7 (14%)	8 (22%)	
Not disclosed	1 (1.2%)	1 (2.0%)	0 (0%)	
Lifetime pregnancies				0.16
2+	59 (69%)	32 (64%)	27 (75%)	
1	6 (7.0%)	2 (4.0%)	4 (11%)	
0	18 (21%)	13 (26%)	5 (14%)	
Not disclosed	3 (3.5%)	3 (6.0%)	0 (0%)	
Lifetime sexual partners				0.079
3+	34 (40%)	22 (44%)	12 (33%)	
1 or 2	40 (47%)	18 (36%)	22 (61%)	
0	8 (9.3%)	7 (14%)	1 (2.8%)	
Not disclosed	4 (4.7%)	3 (6.0%)	1 (2.8%)	
Sexually active				0.40
Yes	56 (65%)	32 (64%)	24 (67%)	
No	27 (31%)	15 (30%)	12 (33%)	
Not disclosed	3 (3.5%)	3 (6.0%)	0 (0%)	
Diabetes status				0.26
T2D	10 (12%)	5 (10%)	5 (14%)	
T1D	4 (4.7%)	1 (2.0%)	3 (8.3%)	
Prediabetic	3 (3.5%)	3 (6.0%)	0 (0%)	
None	69 (80%)	41 (82%)	28 (78%)	
Urogenital symptoms				0.052
Yes	33 (38%)	20 (40%)	13 (36%)	
No	43 (50%)	21 (42%)	22 (61%)	
Not disclosed	10 (12%)	9 (18%)	1 (2.8%)	
Antibiotic use				0.030
Yes	21 (24%)	7 (14%)	14 (39%)	
No	53 (62%)	35 (70%)	18 (50%)	
Not disclosed	12 (14%)	8 (16%)	4 (11%)	
CST				0.76
I	18 (21%)	12 (24%)	6 (17%)	
II	4 (4.7%)	3 (6.0%)	1 (2.8%)	
III	33 (38%)	19 (38%)	14 (39%)	
IV-A	4 (4.7%)	2 (4.0%)	2 (5.6%)	
IV-B	1 (1.2%)	0 (0%)	1 (2.8%)	
IV-C	25 (29%)	14 (28%)	11 (31%)	
V	1 (1.2%)	0 (0%)	1 (2.8%)	

^*a*^ Median (range) or frequency (%).

^*b*^ Wilcoxon rank sum test; Pearson’s Chi-squared test; Fisher’s exact test.

### Alpha diversity and lesser abundant taxa differentiate RA from control vaginal microbiota

**Fig 1:**
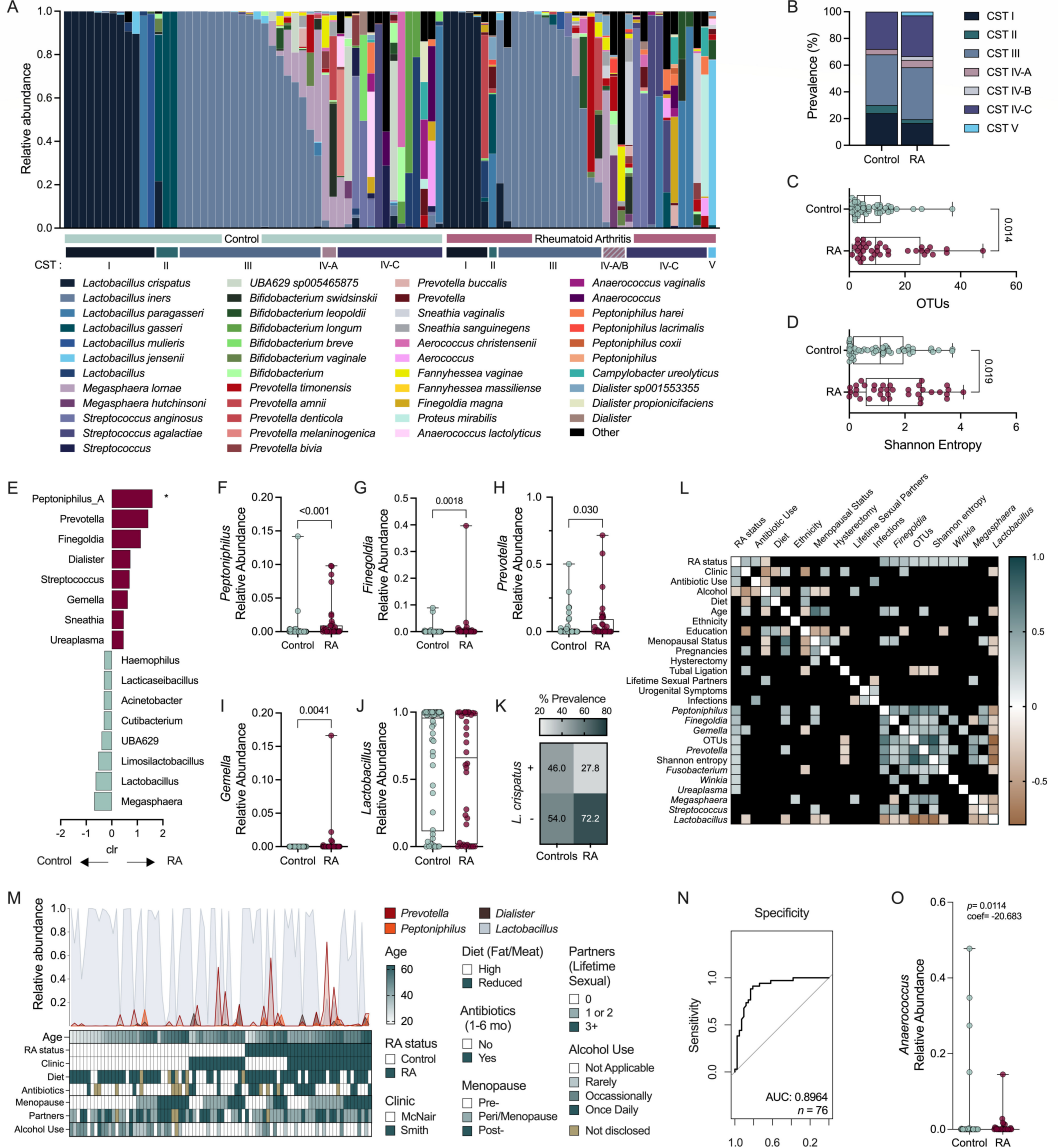
Low abundance taxa distinguish the vaginal microbiota of those with and without RA. Vaginal samples of RA (*n* = 36) and controls (*n* = 50) were subjected to long-read 16S rRNA gene sequencing. (**A**) Relative abundances of the top 15 genera, broken down by species, and their respective community state type (CST). (**B**) CST prevalence in each group. Alpha diversity measured by (**C**) OTUs and (**D**) Shannon entropy. (**E**) ANCOM-determined genera associated with disease state. (**F**) *Peptoniphilus*, (**G**) *Finegoldia*, (**H**) *Prevotella*, and (**I**) *Gemella* relative abundances. (**J**) *Lactobacillus* relative abundance and (**K**) *L. crispatus* prevalence are insignificant between groups. (**L**) Strength of correlations between RA and clinical or microbial variables (unadjusted, non-significant correlations are blacked out). (**M**) Variables included in current and future regression analyses, and the relative abundances of microbes used in (**N**) ROC curve of logistic regression model predicting RA as an outcome of (**M**); (**N**) excludes lifetime sexual partners. (**O**) Relative abundance of *Anaerococcus* in each group. Bars (**A**) and symbols (**C, D, F–J, O**) represent individual subjects. Data were analyzed by Fischer’s exact test (**B,K**), Mann-Whitney U test (**C, D, F–J**), ANCOM (E, significance denoted by *), unadjusted Spearman correlation (**L**), and multiple logistic regression (**N–O**). Regression analyses (**N–O**) were performed on a subset (*n =* 42 controls and *n* = 34 RA post removal of samples missing data on antibiotic use and lifetime sexual partners). Significant *P*-values are shown.

Vaginal microbiomes are grouped into community state types (CSTs) based on the presence of one of four primary *Lactobacillus* species (*L. crispatus*, *L. gasseri*, *L. iners*, and *L. jensenii* as CST I, II, III and V, respectively) (19, 26, 27). In about 25% of women, a non-optimal vaginal microbiome, characterized by a mixture of anaerobic organisms including *Gardnerella* or *Prevotella* spp. (CST IV), is associated with increased risk for adverse gynecological outcomes (19, 26). To determine whether women with RA had altered vaginal microbial compositions, vaginal swab samples were subjected to long-read 16S rRNA gene sequencing and were categorized into CSTs using the VALENCIA classifier (27) (Fig. 1A). CST prevalence was comparable between RA and controls (Fig. 1B). Regarding alpha diversity, the RA cohort displayed elevated OTUs (Fig. 1C) and greater richness and evenness as measured by Shannon Entropy (Fig. 1D). Analysis of compositions of microbiomes (ANCOM) identified differential relative abundant genera, such as *Peptoniphilus*, *Prevotella*, *Finegoldia*, and *Dialister*, associated with RA, and *Megasphaera*, *Lactobacillus,* and *Limosilactobacillus* associated with controls (Fig. 1E). The *Peptoniphilus*, *Finegoldia*, *Prevotella*, and *Gemella* signatures were retained in the RA group, whereas the *Lactobacillus* relative abundance was not significantly associated with the control group when assessed by Mann-Whitney U-test (Fig. 1F through J). Although controls had 2.2 greater odds of *L. crispatus* detection than the RA group, this difference was non-significant (95% CI: 0.87–5.5; *P =* 0.067, Fig. 1K).

Spearman correlation showed RA status was positively associated with use of antibiotics 1–6 months prior and microbial features, including *Peptoniphilus*, *Finegoldia, Gemella, Prevotella, Fusobacterium, Winkia,* and *Ureaplasma* (Fig. 1L). To account for host factors, regression analyses were performed using the variables shown in Fig. 1M and are detailed in Table S1. A covariate adjusted receiver operating characteristic (ROC) curve using backwards selection of input variables (clinic site, diet, antibiotic use, menopausal status, and alcohol use along with relative abundances of *Prevotella*, *Peptoniphilus*, *Dialister, and Lactobacillus*) predicted RA status with an area under curve (AUC) of 0.896 (Fig. 1N). Multiple linear regression revealed that the genus *Anaerococcus* was more abundant in controls (Fig. 1O).

### RA clinical factors correlate with vaginal microbial signatures

**Fig 2:**
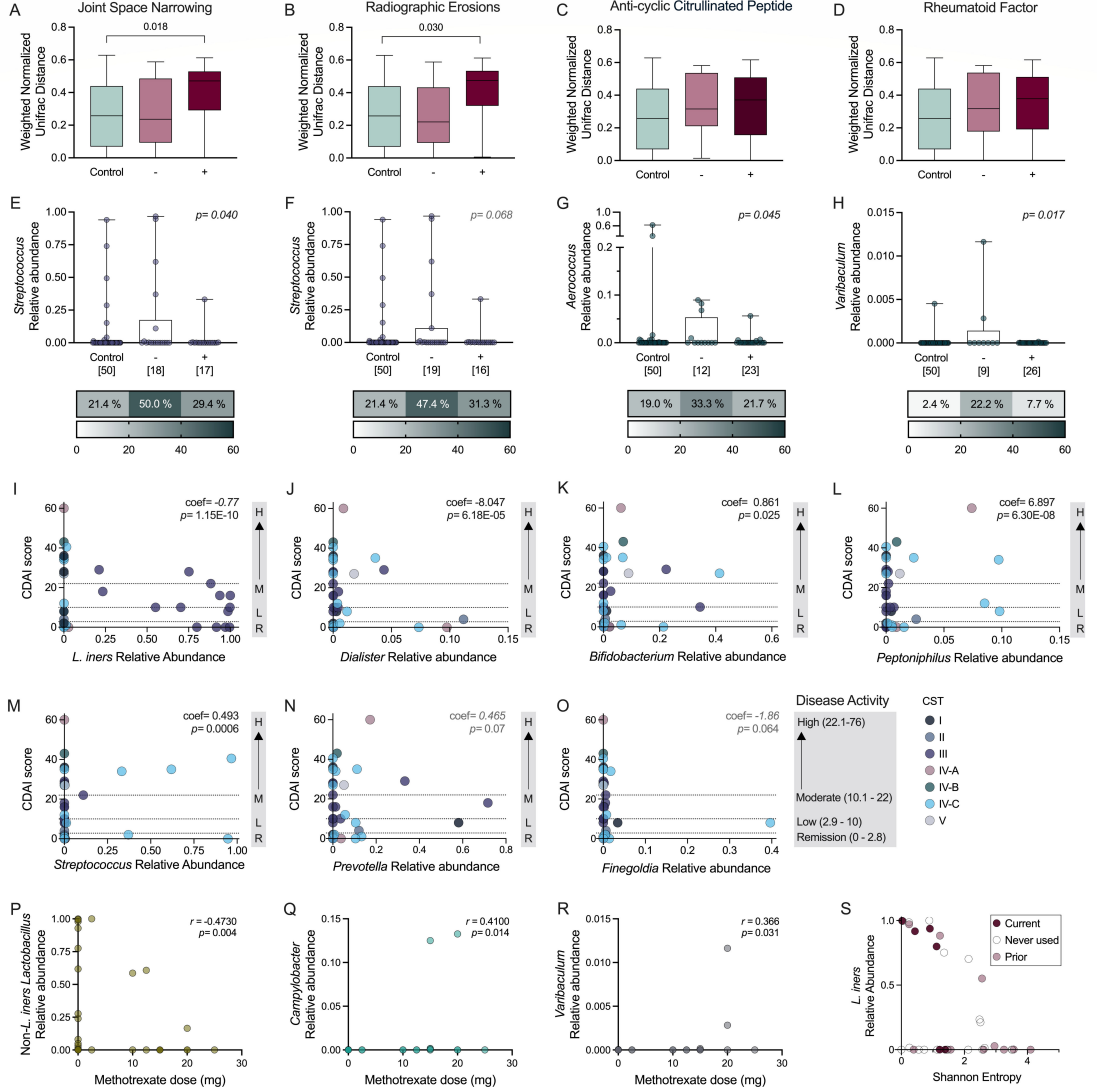
Vaginal microbial signatures differ across RA disease severity metrics. Weighted normalized unifrac distances with binary outcome variables (negative or positive detection) for (**A**) joint space narrowing (JSN) or (**B**) radiographic erosions (RE), and (**C**) serum detection of anti-cyclic citrullinated peptide (ACCP) or (**D**) serum detection of rheumatoid factor (RF) compared to controls (*n* = 35 RA, *n* = 50 controls). Relative abundances and prevalence of taxa associated with (**E**) JSN, (**F**) RE, (**G**) serum ACCP, and (**H**) serum RF as informed by logistic regression analysis with controls shown for reference. Linear regression analysis of CDAI and (**I**) *L. iners*, (**J**) *Dialister*, (**K**) *Bifidobacterium,* (**L**) *Peptoniphilus*, (**M**) *Streptococcus*, (**N**) *Prevotella*, and (**O**) *Finegoldia* (*n* = 35). Dose association between methotrexate and relative abundance of (**P**) non-*L. iners Lactobacillus* spp. and less abundant (**Q**) *Campylobacter* and (**R**) *Varibaculum.* (**S**) Relative abundance of *L. iners* as an interaction between Sulfasalazine (current, prior, and never used) and Shannon entropy within the RA group. Bars (**A–D**) represent Tukey’s box and whisker plots, and symbols represent individual subjects (**E–S**). Sample size is denoted beneath box plots in panels **E–H**. Data were analyzed by PERMANOVA comparing controls and RA subgroups (**A–D**), multiple linear regression determining bacterial abundance as an outcome of positive/negative phenotype or CDAI score (**E–O**), unadjusted Spearman correlation (**P–R**), and linear regression (**S**) between medication and microbial abundance. Prior medication usage (**S**) was combined with never used but remains distinctly colored (*n* = 36). Significant adjusted *P*-values are shown in black, and non-significant *P*-values below 0.1 are shown in gray.

Oral and gastrointestinal microbial signatures are correlated with RA disease activity and symptom flares (10, 28). While bacteria can modulate inflammation directly, microbial communities are also responsive to inflammatory environments (29). To determine whether RA-associated disease parameters are likewise associated with vaginal microbial composition, we assessed correlations between vaginal microbes and radiographic changes or autoantibody presence. Joint space narrowing (JSN) corresponds to cartilage damage and radiographic erosions (RE) to bone destruction (2). RA patients with documented JSN (*n* = 17) or RE (*n* = 16), 15 of whom presented with both JSN and RE (Fig. S2F), had phylogenetically distinct vaginal compositions compared to controls, whereas RA patients without erosive disease (no JSN or RE) were comparable to controls (Fig. 2A and B). No microbial composition differences were seen between controls and RA patients with and without detection of serum anti-cyclic citrullinated peptide (ACCP) antibodies (*n* = 23) and RF (*n* = 26) (Fig. 2C and D; Fig. S2F). Multiple logistic regression determining joint damage or seropositivity within the RA group as an outcome of bacterial abundance detected taxonomic differences (controls depicted for comparison) revealed *Streptococcus* relative abundance was decreased in JSN+ individuals with a non-significant reduction in RE+ individuals (Fig. 2E and F). Despite overall phylogenetic similarity, both ACCP+ and RF+ groups showed reduced *Aerococcus* compared with negative-testing counterparts, and RF+ status was associated with lower *Varibaculum* (Fig. 2G and H).

CDAI reflects current disease activity at the time of sample collection (30). Using multiple linear regression to assess the association between CDAI score and relative abundance of the most prevalent taxa, we observed inverse correlations with *L. iners* and *Dialister,* but positive correlations with *Bifidobacterium, Peptoniphilus,* and *Streptococcus* abundance and CDAI score (Fig. 2I through M). In our cohort, CDAI did not correlate to menopausal stage or time since the most recent onset of menses, suggesting that CDAI and associated microbial signatures were not driven by hormonal patterns (Fig. S2C and D). Other prevalent taxa, *Prevotella* and *Finegoldia,* were not significantly linked to CDAI (Fig. 2N and O). Additional significant correlations are provided in Table S1.

To determine whether disease-modifying anti-rheumatic drugs impact microbial composition in the vaginal tract as in the gut (13), we correlated current medication dosage/usage and microbial relative abundances. Biologics (monoclonal antibodies) were not assessed against microbial composition given their infrequent use in the cohort. Methotrexate dose, the most common medication in our cohort (Fig. S1), was inversely correlated with non*-L.iners Lactobacillus* and positively correlated with low abundance, low prevalence taxa *Campylobacter* and *Varibaculum* (Fig. 2P through R) via Spearman’s correlation. Sulfasalazine positively correlated with relative abundance of *L. iners,* a phenomenon more pronounced when considering the interaction between the medication and overall composition (Fig. 2S). Prednisone dose and hydroxychloroquine use did not impact any genera.

### Specific vaginal cytokines are elevated in RA, and levels are associated with diet and menopausal status

**Fig 3:**
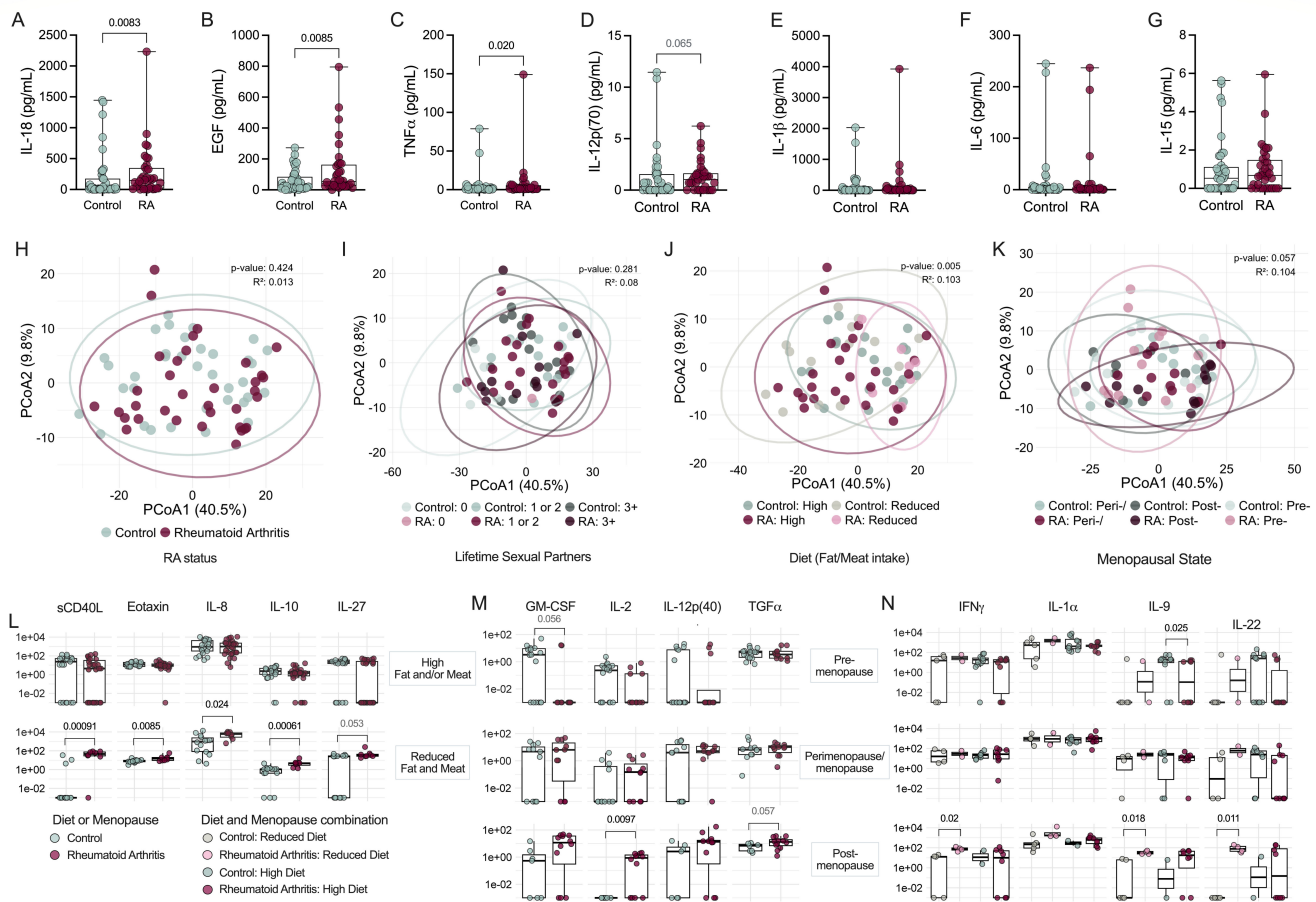
RA is associated with increased vaginal inflammation when stratified by menopausal and dietary factors. Vaginal swab samples were analyzed using a 48-plex ELISA. Unadjusted comparisons of differential (**A**) IL-18, (**B**) EGF, and (**C**) TNF, and non-differential (**D**) IL-12p70, (**E**) IL-1β, (**F**) IL-6, and (**G**) IL-15 in vaginal fluid of control (*n* = 41) and RA subjects (*n* = 36). Overall immune profiles clustered by (**H**) RA status and stratified by (**I**) history of lifetime sexual partners, (**J**) diet described by either high fat and/or meat intake (high) or both reduced fat and meat intake (reduced), and (**K**) menopausal status (*n* = 35/group). Immunological markers differentially expressed in the RA group when stratifying by (**L**) diet, (**M**) menopausal status, and a combination of (**N**) diet and menopausal status. Symbols represent individual subjects. Data were statistically analyzed by Mann-Whitney U test (**A–G, L–N**) or PERMANOVA (**H–K**). Multi-linear regression analyses are provided in Table S1. Significant adjusted *P*-values are shown in black, and non-significant *P*-values below 0.1 are shown in gray.

Microbial signatures corresponding with RA could potentially cause, or derive from, local inflammation (29, 31). To determine whether those with RA had elevated vaginal inflammation, we performed 48-plex cytokine analyses of vaginal samples. Interleukin (IL)-18, epidermal growth factor (EGF), and tumor necrosis factor (TNF) were significantly higher in RA samples, with a non-significant increase in IL-12p70 and no variation in other cytokines, including RA-associated IL-1β, IL-6, or IL-15 (Fig. 3A through G). However, neither RA status alone nor lifetime sexual partners, which was nearly disproportionate between groups, explained the variance among vaginal immune profiles (Fig. 3H and I; Table 1). Additional variables, including ethnicity, history of hysterectomy or tubal ligation, contraceptive use, antibiotic use, or recent sexual activity could not explain variances in vaginal inflammation (Table S1). Rather, diet and menopausal status had greater potential to differentiate cytokine distributions in the context of RA (Fig. 3J and K). The greatest cytokine drivers of clustering differences were MIP-1β, IL-1β, TNFα, IL-8, and IL-2 along axis PCoA1, whereas PCoA2 was driven by IL-12p40, MIP-1β, IL-6, INFα, and RANTES (Table S1).

When stratified by diet (fat/meat intake), sCD40L, eotaxin, IL-8, IL-10, and IL-27 were increased in RA patients compared with controls in individuals with reduced fat/meat consumption (Fig. 3L). When stratified by menopausal status, granulocyte-macrophage colony-stimulating factor (GM-CSF), IL-2, IL-12p40, and transforming growth factor alpha (TGFα) were elevated in post-menopausal RA versus control samples (Fig. 3M). When stratified by both diet and menopausal status, interferon (IFN)γ, IL-1α, IL-9, and IL-22 were elevated in post-menopausal, reduced fat/meat diet RA patients compared with matched controls (Fig. 3N). Pre-menopausal RA patients on high fat/meat diets displayed decreased IL-1α and IL-9 compared with controls (Fig. 3N). Given that inflammatory cytokines fluctuate throughout the menstrual cycle (32), pre-menopausal women with paired cytokine data were stratified by RA status and proximity to menstruation (Fig. S1C and S3A through H). Of the eight cytokines linked with RA and menopausal status (Fig. 3M and N), GM-CSF was the only one that significantly fluctuated and was higher in controls during timeframes corresponding with the luteal phase compared with the follicular phase (Fig. S3D).

Vaginal microbes were also assessed in relation to local immune mediators via Spearman correlation and PCoA clustering (Table S1). Although unimpacted by medication use, IL-18 levels positively correlated with RA-associated *Prevotella*, *Peptoniphilus,* and *F. magna*, and inversely correlated with *L. crispatus* (Fig. S3I through K). TNF was associated with *Streptococcus* and *Prevotella*, whereas EGF was not significantly correlated with microbial features (Fig. S3J and K). Samples were also grouped by *Lactobacillus* dominance in the control and RA cohorts or by overall CST regardless of RA status. Neither explained variance in cytokine composition (Fig. S3L and M). While batch effect seemed to have influence on clustering across PCoA2, those with “High” fat and meat intake were significantly more represented in the first batch and may explain the apparent variance in cytokine profiles across batches (Fig. S3N; Table S2). Prediction of RA status using bootstrapping of a logistic regression model combining *Prevotella*, *Peptoniphilus*, EGF, the interaction between sCD40L and diet, menopausal status, and clinic resulted in a mean AUC of 0.97 comparing 34 RA to a subset of 13 vaginal ACPA negative controls (95% CI [0.90, 1], Fig. S4A and B). To test the model’s performance, we used the same method comparing all 35 RA to the remaining 22 controls (vaginal ACPA positive or unknown) which produced an AUC of 0.93 (95% CI [0.86, 0.99]) indicating reproducibility (Fig. S4C and D).

### Vaginal cytokine levels are correlated with RA clinical factors

**Fig 4:**
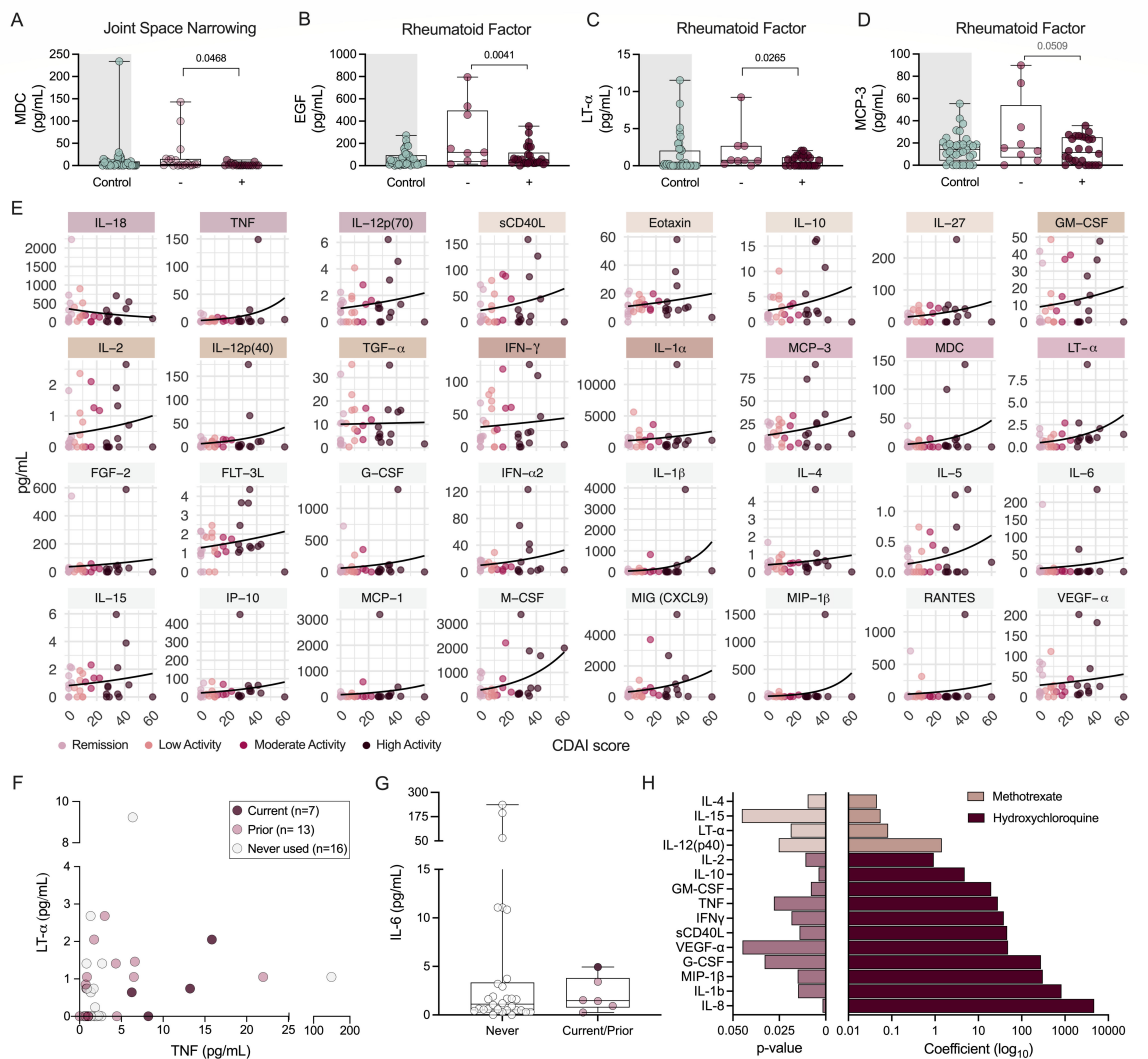
Elevated inflammatory markers correlate with increased CDAI score but not morphologic or serologic RA manifestations. Distributions of (**A**) MDC based on JSN status (*n = 18* per group*)* and (**B**) EGF, (**C**) LT-α, and (**D**) MCP-3 based on serum RF positivity status (*n* = 26 RF positive, *n* = 9 RF negative). Controls are depicted as a reference. (**E**) Cytokines predictive of CDAI score when accounting for diet and menopausal bins. Use of (**F**) TNF and (**G**) IL-6 inhibitors and their corresponding concentrations in vaginal samples. (**H**) *P*-values and coefficients of methotrexate dose and hydroxychloroquine use in predicting cytokine concentration. Symbols represent individual subjects (**A–G**). Immune factors colored by RA association: control versus RA, diet subgroups, menopausal subgroup, diet: menopausal subgroup, and JSN/RF detection. Data were analyzed by logistic regression for positive and negative detection of factors within the RA group, accounting for diet and menopausal status (**A–D**), non-linear regression using non-adjusted Poisson distribution with 95% CI (**E**), or multiple logistic regression predicting medication use as a factor of cytokine concentrations (**F–G**). Prior use colored for reference but combined with (**F**) never used or (**G**) current group. (**H**) Multi-linear model predicting cytokine as an outcome of medication use when adjusted for menopausal status, clinic, and diet. Multi-linear regression analyses are provided in Table S1. Significant *P*-values are shown in black, and non-significant *P*-values below 0.1 are shown in gray (**A–D, F–H**).

Multiple studies demonstrate elevated circulating inflammatory cytokines in RA patients with active disease versus those in remission/quiescence (33, 34). To assess whether vaginal cytokine signatures correspond with disease metrics, we performed adjusted logistic regression analyses within the RA cohort (Table S1). Vaginal macrophage-derived chemokine (MDC) levels were decreased in women with JSN (Fig. 4A), whereas detection of serum RF was associated with decreased EGF, lymphotoxin (LT)-α, and monocyte chemotactic protein (MCP)-3 (Fig. 4B through D). No associations between radiographic erosions or detection of serum ACCP antibodies were observed with vaginal cytokines.

When assessing CDAI and vaginal cytokine associations, interestingly, IL-18 was the only cytokine inversely correlated with disease activity (Fig. 4E). Thirty-one additional cytokines positively correlated with CDAI using a Poisson distribution model adjusted for diet and menopausal status. With the exception of EGF, IL-8, IL-9, and IL-22, 16 of the cytokines related to RA diagnosis in association with diet, menopausal status, or both were also correlated with CDAI (Fig. 3 and 4E). The other 16 cytokines were uniquely linked to CDAI (Fig. 4E). Higher *Prevotella* abundance correlated with 6 CDAI-associated immune markers, and species specificity within *Peptoniphilus* was demonstrated by the positive correlation of 6 markers with *P. hare*i but negative correlation of *P. lacrimalis* with 12 other markers (Fig. S3J and K).

To observe whether biologics impact vaginal cytokines, we assessed anti-TNF (adalimumab, certolizumab pegol, etanercept, golimumab, and infliximab) and anti-IL-6 (tocilizumab and sarilumab) medication use and their corresponding vaginal cytokines. Vaginal TNF and LT-α (a TNF family cytokine that binds the same receptor) concentrations did not differentiate patients currently taking anti-TNF medication from those with prior or no history of use (Fig. 4F). Due to small numbers, current and prior anti-IL-6 users were assessed together against non-users, and no medication-mediated vaginal IL-6 suppression was detected (Fig. 4G). Women taking methotrexate exhibited slight, but significantly increased, IL-4, IL-15, LT-α, and IL-12p40 (Fig. 4H). Hydroxychloroquine use was associated with increased levels of 11 cytokines (Fig. 4H).

### Vaginal ACPA predominantly correlates with *Streptococcus* and inflammatory signatures in RA

**Fig 5:**
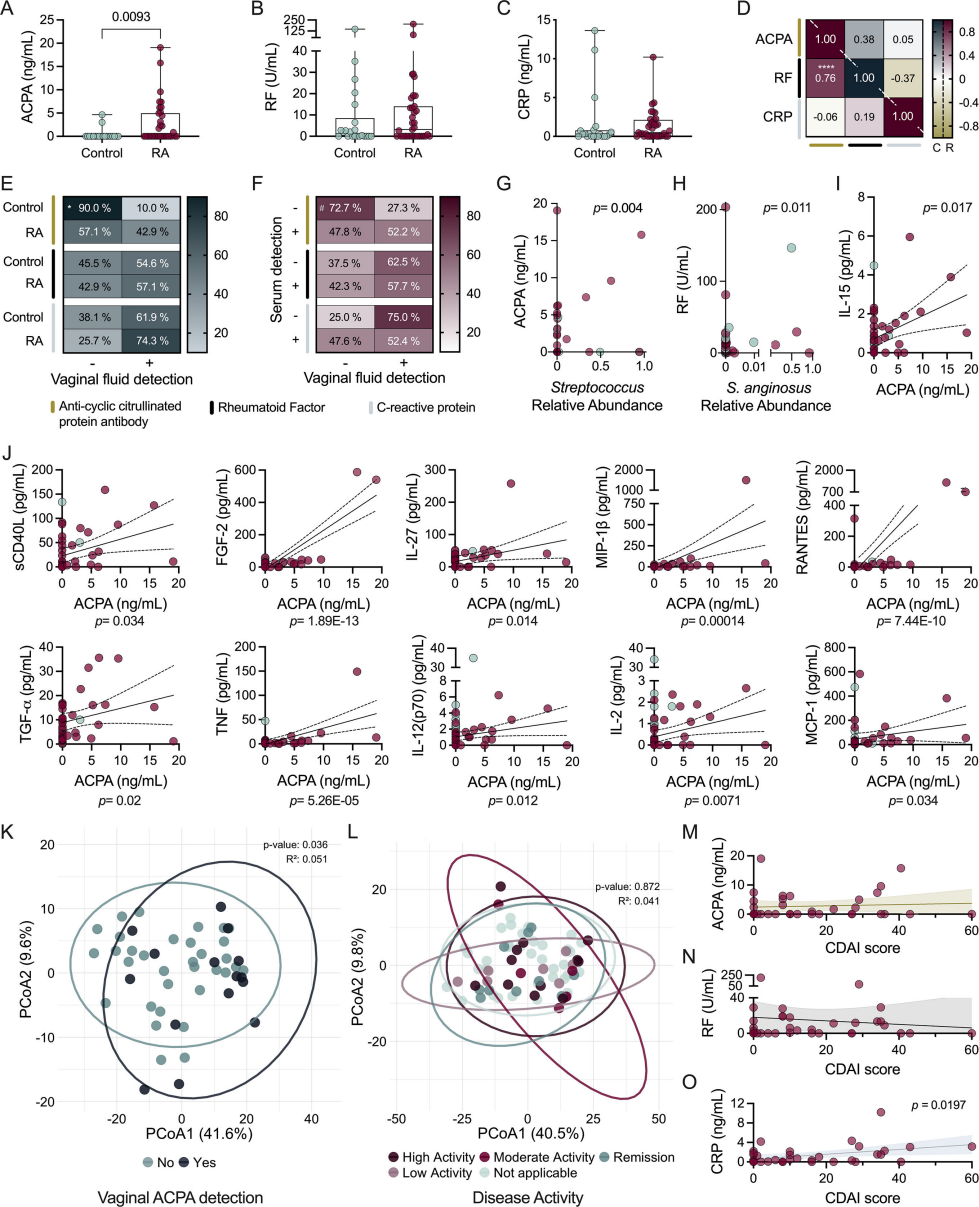
RA biomarkers are detected in the vaginal tract and ACPA levels correlate with elevated inflammatory cytokines predominantly in the RA cohort. Vaginal concentrations of (**A**) anti-citrullinated protein antibodies (ACPA), (**B**) rheumatoid factor (RF), and (**C**) C-reactive protein (CRP) (*n* = 35 RA, *n* = 20–22 controls). (**D**) Correlated detection of vaginal ACPA, RF, or CRP in controls (“C”, upper right) and RA cohort (“R”, bottom left). (**E**) Prevalence of ACPA, RF, or CRP in control and RA vaginal samples. Within individuals of the RA cohort, (**F**) co-detection of serum ACCP antibodies and vaginal ACPA, serum and vaginal RF, and serum and vaginal CRP. Biomarker concentrations dependent on microbial interactions with RA: (**G**) vaginal ACPA with *Streptococcus* spp. and (**H**) vaginal RF with *S. anginosus*. Correlations between vaginal ACPA concentrations and cytokines also associated with *Streptococcus* abundance in (**I**) both control and RA and (**J**) only RA groups. Overall immune profiles clustered by (**K**) vaginal ACPA presence regardless of RA status and (**L**) disease activity index in the RA cohort. Correlations between vaginal (**M**) ACPA, (**N**) RF, and (**O**) CRP concentrations and CDAI score. Symbols represent individual subjects. Spearman correlations and 95% CI are shown for immune markers found significant in multiple linear regression analysis (**G–J, M–O**). Data were analyzed by Mann-Whitney U test (**A–C**), Spearman correlation (**D**), Fisher’s Exact test (**E**), McNemar’s test (**F**), multiple linear regression (**G–J, M–O**), and (**K and L**) PERMANOVA. Significant *P*-values shown (**A–C, G–M**), or indicated as: ^#^*P* = 0.061, *P = 0.015, ****P < 0.001 (**D–F**).

Serum ACCP antibodies and RF inform RA diagnosis while CRP captures inflammation; however, the latter two are non-RA-specific (35–37), and all three can be induced by microbially-mediated inflammation (35, 38). We quantified vaginal anti-citrullinated protein antibodies (ACPA), RF, and CRP levels via ELISA (Fig. 5A through C). ACPA was elevated in RA patients with no differences observed in RF levels despite co-occurrence in the RA, but not the control, group (Fig. 5A, B and D). ACPA was detected at higher frequency within the RA cohort (OR = 6.750), while RF could be detected in about half of all women and CRP in more than half of all women (Fig. 5E). Neither ACPA, RF, nor CRP differed between the control and RA groups at any given menopausal stage (Fig. S5A through C). Of premenopausal RA samples, no correlations were found between concentrations of the three biomarkers and time elapsed since the start of the most recent menses (Fig. S5D through F). In the control group, however, RF levels were positively correlated with time since the most recent menstrual cycle (Fig. S5G through I).

Within the RA group, serum and vaginal detection of ACPA (specifically ACCP antibodies in serum) or RF were not interrelated (Fig. 5F), limiting the potential of vaginal levels to serve as a proxy for serum ACCP antibodies and RF (38). Within the RA group, vaginal ACPA and RF positively correlated with *Streptococcus* and *S. anginosus*, respectively (Fig. 5G and H). IL-15 positively correlated with vaginal ACPA levels in both groups (Fig. 5I). Within the RA group, twenty-one additional cytokines were positively correlated with vaginal ACPA levels, ten of which positively correlated with *Streptococcus* abundance (Fig. 5J; Fig. S3J, K and S6A). Several of these had overlapping positive correlations with RF including FGF-2, RANTES, G-CSF, and IL-6, with positive correlations for FGF-2 also observed in the control group (Fig. 5J; Fig. S6A and B). Overall, vaginal ACPA was the only biomarker significantly associated with differential clustering of immune mediators (Fig. 5K; Table S1). Furthermore, clustering of vaginal immune profiles was independent of disease activity (Fig. 5L), and neither vaginal ACPA nor RF was correlated with CDAI despite shared cytokine associations (Fig. 5M and N).

CRP was detected in the majority of RA and controls irrespective of genitourinary symptoms or vaginal ACPA and RF (Fig. 5C through E; Fig. S6C and D). Consistent with prior studies, vaginal CRP detection and concentrations did not reflect systemic CRP values(36, 37) (Fig. 5F; Fig. S6E). Microbially, CRP was associated with *L. paragasseri* relative abundance (Fig. S6F; Table S1). Interestingly, vaginal CRP positively correlated with CDAI (Fig. 5O). CRP correlated negatively with IL-9 in both RA and control groups and positively with PDGF-AA and VEGF, among others, in the control group only (Fig. S5G).

## DISCUSSION

Rheumatoid arthritis is a multisystemic disease that can lead to irreversible joint damage and decreased quality of life. Despite microbial ties to RA (39) and clinical associations between RA, infertility, and adverse reproductive outcomes (40–42), the vaginal environment has not been described in the context of RA. Our study determined that the RA vaginal microenvironment is distinguished by higher alpha diversity, increased presence of non-optimal taxa such as *Prevotella* and *Peptoniphilus*, and higher levels of growth factors and inflammatory cytokines particularly in postmenopausal stages and diet subsets. Importantly, ACPA was significantly higher in RA vaginal samples, correlating with *Streptococcus* and multiple inflammatory cytokines irrespective of overall disease activity. Vaginal RF and ACPA levels correlated within the RA cohort, but not the controls, strengthening an association between these vaginal markers and RA. Yet, the discordance between vaginal and serum levels of these same factors suggests divergence between systemic and vaginal autoimmune features.

A recent study found that women with systemic lupus erythematosus (SLE) had vaginal microbiomes characterized by increased Shannon diversity, *Peptoniphilus,* and *Streptococcus* compared with controls (43). Similarly, we found that women with RA had higher alpha diversity and *Peptoniphilus*, which has been linked to osteoarticular infections and bacterial vaginosis (44), and women with higher levels of vaginal ACPA had increased relative abundance of *Streptococcus*. Consistent with the relationship between *Prevotella* and RA across mucosal sites, we show *Prevotella* enrichment in the RA vaginal tract, a potential set-up for inflammation or bacterial vaginosis (31, 45). Yet, RA is also linked to rare gut and oral microbes absent from the vaginal tract (11, 12, 46), and taxonomic associations are not consistent across autoimmune diseases: the RA cohort did not demonstrate significantly decreased *Lactobacillus* or increased *Bifidobacterium* as reported in SLE. Furthermore, RA patients on higher methotrexate doses had lower levels of health-associated *Lactobacillus* spp. and women on sulfasalazine were more likely to have higher non-optimal *L. iners* abundance. Together, these taxonomic alterations suggest women with RA may have a higher risk of colonization with vaginal taxa associated with negative gynecologic outcomes.

Elevated vaginal EGF was a consistent RA signature irrespective of menopausal status, diet, microbial profile, CDAI score, or vaginal ACPA. EGF induces estrogen production and mediates estrogen-like effects to induce vaginal epithelial growth (47). Through engaging the EGF receptor, EGF downregulates TNF-induced apoptosis and necroptosis. This presents a dual role for EGF in promoting tissue regeneration and suppressing inflammation, while also driving problematic proliferation of synovial fibroblasts and osteoclasts in RA joints (48). Relatedly, reduced vaginal MDC levels in JSN+ individuals contrast with elevated MDC in damaged synovial joints (49). Vaginal IL-18, known to recruit neutrophils in response to infection (47, 50), was associated with eight microbial features uniquely in RA, suggesting an important role in host-microbe dynamics. Although RA vaginal samples demonstrated elevated inflammation overall, these signatures were distinct from iconic, pathogenic inflammation in RA serum or synovial joints.

CDAI yielded the most microbial and immunologic correlations within the RA cohort. However, lack of correlation between CDAI and ACPA suggests independent mechanisms for immune associations. Furthermore, the lack of associations between menopausal or menstrual stage and RA-associated immune modulators, CDAI, or ACPA supports that differences between control and RA samples or within RA samples are not closely attributed to hormonal stages. ACPAs are generated in response to peptide citrullination via host or bacterial peptidylarginine deaminase (PAD) enzymes or citrullinated bacteria themselves (5, 6, 10). Vaginal autoantibody correlations with bacterial vaginosis further implicate local ACPA production via vaginal microbes such as *Streptococcus* (5, 6, 10, 51–54). Although Streptococci themselves do not express PADs, they can be citrullinated by neutrophil or bacterial PADs to activate ACPA-producing B cells (10). While vaginal detection of CRP and ACPAs has been previously reported (37, 52), this is the first report of vaginal RF to our knowledge. Vaginal ACPAs and RF co-occurred in most RA patients, yet no microbes and only four cytokines were simultaneously associated with ACPA and RF. These data suggest a complex RA vaginal autoimmune environment influenced by CDAI and ACPA status that requires further experimental study to delineate shared and independent mechanisms.

Several aspects of our study design limit interpretation. Despite broad demographic features captured in our cohort, both clinics are located in a single medical center under the same providers. Model overfitting remains a possibility given our small sample size, particularly in microbial analyses where taxonomic presence is variable. Logistic regression, linear regression, and PERMANOVA analyses frequently returned low R-squared values and must be interpreted with caution. Certain variables, such as hormonal contraceptive or antibiotic use, could not be further evaluated due to low sample numbers. Stratification by menstrual stage also subjected analyses to smaller group sizes. Additional limitations include lack of viral and fungal analyses (9) and the broad nature of the questionnaire, which restricted further categorization of diet and reporting of diabetes, autoimmune disease, and other medical conditions in the control group.

We describe correlations within the RA vaginal microbial and immunological landscape, although the directionality of these associations remains unknown. Despite comparable presence of CSTs, taxa, and urogenital symptoms between controls and RA, the functional capacity and immunogenicity of microbes may be different given the unique RA immune profiles. The potential for acquired functions in different stress environments (29) is exemplified by the expression of an additional glycosyltransferase in *L. crispatus* strains from non-*Lactobacillus*-dominant versus *Lactobacillus*-dominant communities (55). Likewise, longitudinal studies and larger cohorts are needed to assess infection risk and the impact of medication and RA flares on vaginal microbial composition. Despite the absence of vaginal and systemic immune correlations during bacterial vaginosis (56), our work compels further study of whether local vaginal immune dynamics instigate systemic changes or vice versa.

Ultimately, understanding the vaginal microbial and immune composition within RA will help inform patient care, monitoring, and risk assessment. Considering the shared impact of menopause-induced hormonal changes on the vaginal microbiome and RA pathogenesis (15), and clear delineation of vaginal immune markers in post-menopausal RA and control patients, post-menopausal women may benefit the most from future vaginal intervention. If vaginal ACPA production contributes to disease onset, prophylactic treatment targeting specific taxa (e.g., *Streptococcus*) could aid in postponing or, ideally, preventing disease onset. If RA and/or RA medications are the driver of increased vaginal microbial alpha diversity, then reduction of inflammation or change in medication may ameliorate vaginal risk profiles in sexually active women (13). Addressing the vaginal environment as a potential site to suppress RA progression, disease activity, and RA-associated sequelae holds promise in improving the quality of life for women living with RA.

## MATERIALS AND METHODS

### Study patient demographics

We recruited women between 18 and 63 years of age from general care and rheumatology outpatient clinics in two hospitals in the Texas Medical Center, Houston, Texas: Baylor Medicine (“clinic 1,” May 2021 through February 2024) and Harris Health Smith Clinic (“clinic 2,” December 2023 through June 2024). Our study includes women clinically diagnosed by a rheumatologist as having seropositive or seronegative RA. Controls were identified by frequency matching on age (±5 years) and menopausal, educational, and type two diabetes status, with all four factors aligning between most individual comparisons (Table 1). Exclusion criteria consisted of active vulvo-vaginal herpes lesions, original diagnosis of juvenile idiopathic arthritis, active pregnancy, known HIV positivity, history of medical problems that would otherwise impact sample collection, and antibiotic use within the last month. Eight RAs and four controls did not disclose antibiotic use but were retained in the study.

### Clinical sample collection

Subjects self-reported dietary and health practices through a study-specific questionnaire provided in English or Spanish. Dietary components carbohydrates, meat, fat, and vegetables were rated on a scale from 1 to 4 or denoted as rarely or never consumed. Samples were then binned into “High Fat or Meat” or “Reduced Fat and Meat” groups. Descriptions of the control and RA cohorts, including self-disclosed supplement and dietary intake, can be found in Fig. S1A. For the RA cohort, clinicians additionally reported current and prior medication use, radiographic and serological history, current CDAI score, and known gynecologic symptoms. RA-specific data is shown in Fig. S2. Vaginal swabs were self-collected by patients instructed to rotate the swab four times clockwise and four times counterclockwise along the vaginal wall, repeated for a total of two swabs. Patients undergoing routine pap smears in clinic 2, including one RA patient, had samples collected by the clinician. Swabs were transported to the laboratory in a chilled anaerobic container, resuspended in 2 mL of sterile phosphate buffered saline (PBS), and stored at −20°C.

### DNA extraction, 16S rRNA sequencing, and analyses

DNA extractions were performed using a Quick-DNA Fungal/Bacterial Microprep Kit (Zymo Research) per manufacturer protocol except for a 15-minute bead-beating step and elution in 40 µL molecular grade water. Long-read 16S rRNA genes were amplified via a 35-cycle PCR with barcoded 27F and 1492R primers, the HotStarTaq Plus Master Mix Kit (Qiagen), and purified using Ampure PB beads (Pacific Biosciences). Sample libraries were prepped using SMRTbell libraries (Pacific Biosciences) and sequenced by MR DNA Lab (Shallowater, TX, USA) on the PacBio Sequel using the diversity assay bTEFAP LONG HIFI 5k, following the manufacturer’s guidelines. Secondary analysis consisted of Circular Consensus Sequencing, using PacBio’s CCS algorithm. Barcodes were removed prior to downstream analysis. Samples were run across seven batches with three of the initial batches (24 swabs, 26% samples) comprising solely of control swabs from one clinic, and subsequent runs consisted of 41%–75% RA samples (Table S3). At least one swab blank was included in each of the seven batches. A second swab blank was present in batches with more than 10 samples, and a kit blank was sequenced in three individual runs that summed 58% of the total samples. Batch effect was considered in the decontam process to elucidate contaminants. Taxa were then removed across all batches.

R package Decontam (57) (R v4.4.2 (2024-10-31) -- “Pile of Leaves”) identified 12 Feature IDs for removal at a threshold of 0.2. Contaminant *Pseudomonas veronii* was manually removed. Reads were denoised using DADA2 with parameters --p-max-ee 2, --p-trunc-q 2, --p-min-len 1000, and --p-max-len 1600 using QIIME2 v2023.5 (58). Operational taxonomic units (OTUs) were assigned using the Greengenes2 reference tree (59). Alpha diversity (OTUs and Shannon), beta diversity (weighted normalized unifrac distance and PERMANOVA), and differential abundance (ANCOM) tests were carried out in QIIME2 (60). Output files were exported and analyzed in R Studio v2024.12.0+ using packages phyloseq v1.50.0 (61), gtsummary v2.1.0, dplyr v1.1.4, plyr v1.8.9, ggpubr v0.6.0, lmtest v0.9.40, tidyverse v2.0.0, car v3.1.3, boot v1.3.31, pROC v1.18.5, and nlme v3.1.168. Community state types (CSTs) were assigned using the VALENCIA classifier retaining *Lactobacillus* spp., *Gardnerella* spp., *Prevotella* spp., *Atopobium* spp., *Sneathia* spp., and *Mobiluncus* spp. names and reassigning all other taxa to their genus or higher level taxonomic name (27). Data visualization was performed using ggplot2 (62) and GraphPad Prism v10.2.3 (GraphPad Software, Inc.). For linear and logistic regression models, only species that were present in >6% of samples were assessed for correlation. In the RA group only, taxa that were present in at least three samples were prioritized for visualization.

### Cytokine quantification and analyses

Vaginal samples underwent a maximum of three freeze-thaw cycles prior to cytokine analyses. Tubes were centrifuged at 10,000 rcf for 15 min prior to being run on a 48-plex MILLIPLEX Human Cytokine/Chemokine/Growth Factor Panel A Magnetic Bead Panel (Cat#: HCYTA-60K-PX48). PBS was used as the matrix solution for standards and controls, following manufacturer instructions. The instrument passed calibration using the Bio-Plex Calibration Kit (Cat#: 171-203060) and passed validation using the Bio-Plex Validation Kit 4.0 (Cat#: 171-203001).

A Logistic-5PL regression type was used to generate analyte standard curves. Cytokines with a zero or negative FI-background value were deemed out of range and assigned a concentration of 0.001. Of 48 cytokines tested, Fractalkine, IL-3, IL-7, IL-13, IL-17A, IL17-E/IL-25, IL-17F, MIP-1α, and PDGFF-AB/BB were undetected in at least 55% of total samples and were excluded from analyses. GRO-α was excluded because of incongruities between runs. No normalization was conducted as all other concentrations were comparable between runs, and total protein in vaginal fluid varies widely (63). Raw values are provided in Table S4**.**

### Statistics

Single-timepoint vaginal swabs are represented by individual symbols on all plots. Contingency and frequency data were analyzed by Fisher’s Exact test. Microbial, cytokine, or protein comparisons were analyzed by two-tailed Mann-Whitney U test. Differential taxa were identified via ANCOM. PERMANOVAs were performed on Euclidean distance matrices. Subsequent pairwise comparisons and dispersion tests using PERMDISP were applied to comparisons where PERMANOVA returned a significant *P*-value. Post-hoc tests were performed using false discovery rate (FDR) and are detailed in Table S1. Two-tailed Spearman correlations were performed between microbial and metadata factors and between microbes and immune factors. Multiple linear, non-linear and logistic regression models incorporated variables that were significantly different in groups (i.e., antibiotic use and lifetime sexual partners) and are denoted in Table S1. Menopausal stage and dietary groups were always included, and serum ACPA detection was added to within-RA analyses (except when assessing RF and CDAI because of collinearity). Bacteria included in logistic regression models (Fig. 1) were determined using the backwards deduction method. Cytokine and ACPA-pertaining models included stepwise addition of significant variables, choosing the best model based on lower AIC and fewest variables. Coefficients and *P*-values for significant regression model variables are provided in Table S1. Variable coefficients and adjusted *P*-values are shown unless otherwise stated. Mann-Whitney U test *P*-values are shown for comparisons significantly identified by regression analysis. Kruskal-Wallis with Dunn’s multiple comparisons test was used in comparisons with positive or negative detection of morphologic or serum markers. All data were considered non-parametric and non-paired. *P*-values < 0.05 were considered statistically significant.

## Data Availability

Sequencing data used in this study are available in EBI under the accession number PRJEB88521. Scripts are accessible at GitHub under project “Patras2025-RA_vaginal_microenvironment.”

## SUPPLEMENTAL MATERIAL

Supplemental material spectrum.03602-25-s0001.pdf Figures S1 to S6, Tables S1 to S4.

Table S1 spectrum.03602-25-s0002.xlsx This table contains all regression models used in the manuscript, stratified by individual comparisons and data sets, with additional statistical parameters and significant discoveries indicated in separate columns.

Table S4 spectrum.03602-25-s0003.csv This table contains raw values for all cytokines and RA-associated biomarkers at the indicated concentrations in each column.

ASM does not own the copyrights to Supplemental Material that may be linked to, or accessed through, an article. The authors have granted ASM a non-exclusive, world-wide license to publish the Supplemental Material files. Please contact the corresponding author directly for reuse.
