# Supplementary material for: A cross-sectional analysis of the vaginal microenvironment in rheumatoid arthritis
Source: Microbiol Spectr. 2026 Apr 30;14(6):e03602-25. doi: 10.1128/spectrum.03602-25 (PMC13227958; doi:10.1128/spectrum.03602-25)
Supplement: Supplemental material — Figures S1 to S6, Tables S1 to S4. [file spectrum.03602-25-s0001.pdf]

SUPPLEMENTAL MATERIAL

A cross-sectional analysis of the vaginal microenvironment in rheumatoid arthritis

Marlyd E. Mejia, Savannah Bowman, Jessica Lee, Ali El-Halwagi, Keshia Ferguson, Maryjo Maliekel, Yixuan Zhou, Camille Serchejian, Clare M. Robertson, Mallory B. Ballard, Lee B. Lu, Sobia Khan, Olubunmi O. Oladunjoye, Shixia Huang, Sandeep K. Agarwal, and Kathryn A. Patras

Contents:

Supplementary Figures 1-6

Legends for Supplementary Tables 1-4

Supplementary Tables 2 and 3

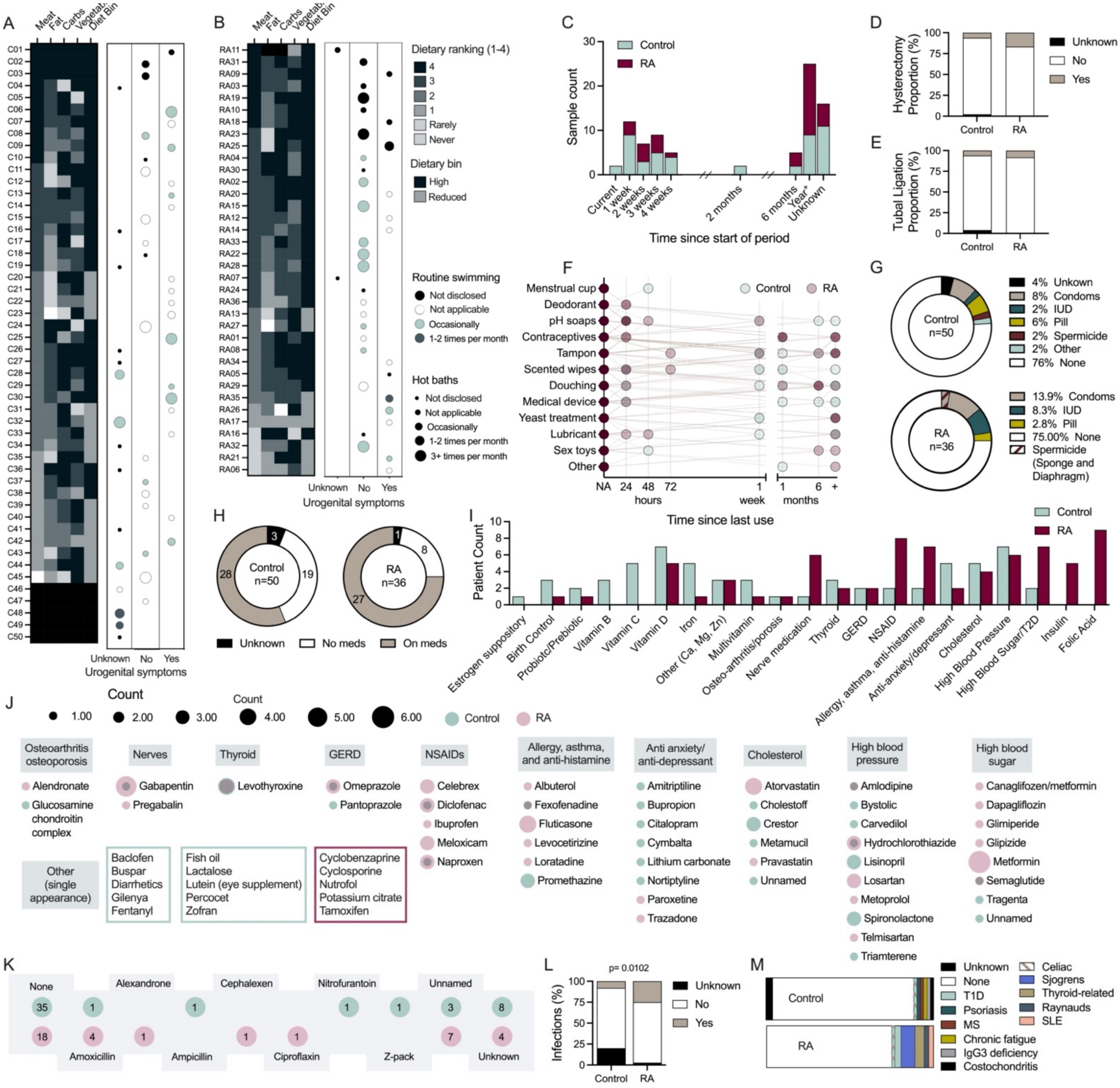

Supplemental Figure 1. Medical information and recent practices of the study cohort. Individual's diet breakdown and diet bin determination, and swimming and bath consistency stratified by presence of urogenital symptoms in (**A**) control and (**B**) RA groups. (**C**) Time since the start of the most recent menstruation. Proportion of women with history of (**D**) hysterectomy or (**E**) tubal ligation. (**F**) Most recent use of products/items inserted into the vaginal canal; increasing intensity signifies multiplicity of use, and purple tones indicate overlap of control and RA use. (**G**) Contraceptive usage. (**H**) Prevalence and (**I**) types of medications or supplements in use. (**J**) Names of medications with overlapping use depicted by overlay of control (teal) and RA (pink) circles. (**K**) Antibiotics in use by control (top) and RA (bottom) individuals. (**L**) Proportion of women with recent infections. (**M**) Additional autoimmune diseases in each group. Data were analyzed by Fisher's exact test (D-E,L-M). Significant *P*-values are shown.

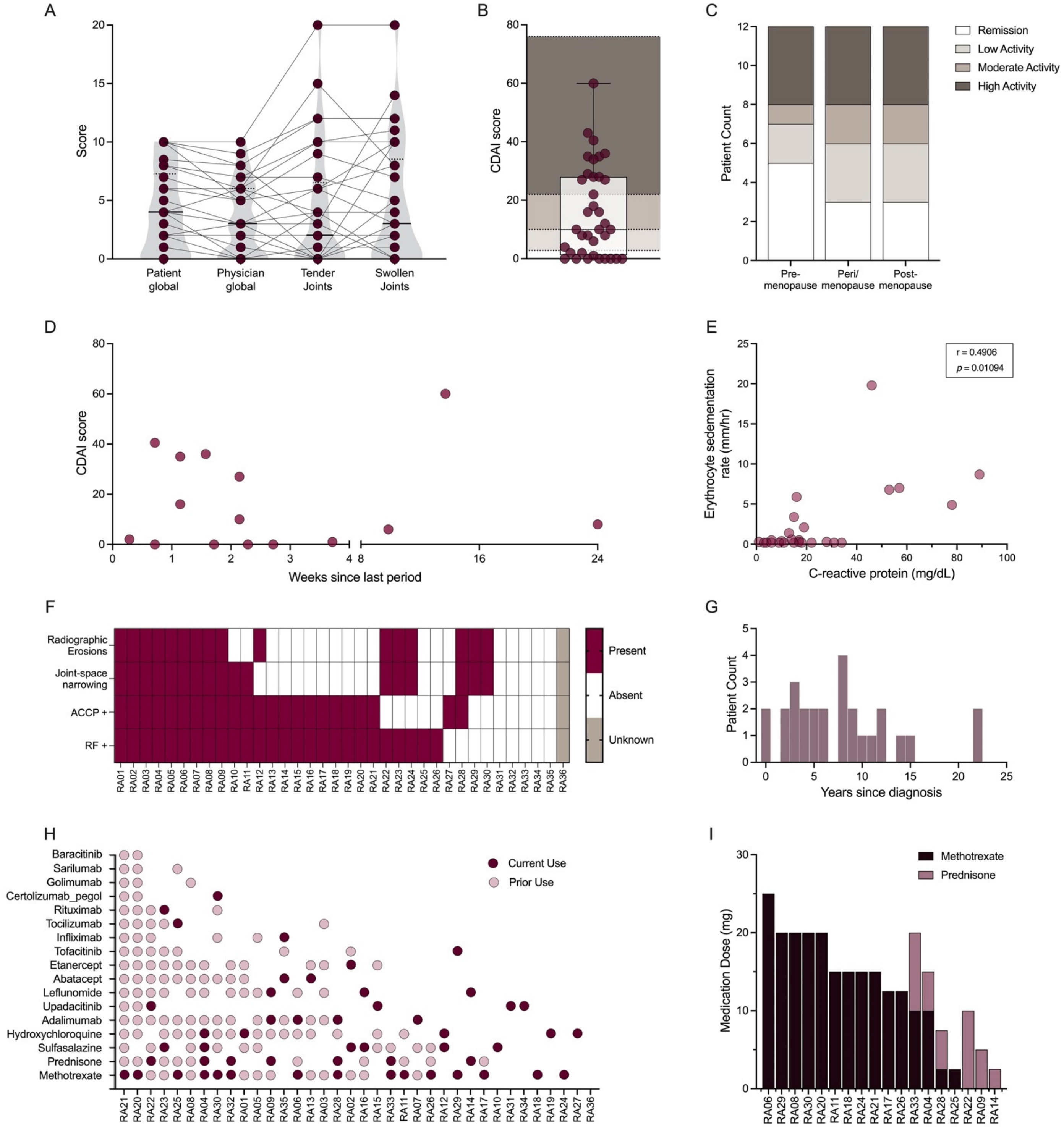

Supplemental Figure 2. Clinical features within the RA cohort. Disease severity was measured by compiling (**A**) patient and physician global scores, and tender and swollen joint counts to determine a (**B**) clinical disease activity index (CDAI) score. CDAI stratification across (**C**) menopausal stages and (**D**) time since onset of most recent menstruation. (**E**) Within-individual erythrocyte sedimentation rate and serum C-reactive protein concentrations. (**F**) Presence of morphological symptoms and serum markers within individuals. (**G**) Duration of disease in years. (**H**) Medication history and (**I**) current dose prescribed for methotrexate and prednisone in current users (one dose not known). Symbols represent individuals (A-C) with lines connecting individuals across clinical factors (A). Columns represent one person (D, F, G). Data were analyzed by Fischer's exact test (C), Spearman correlation (D,E) with *P*-value indicated.

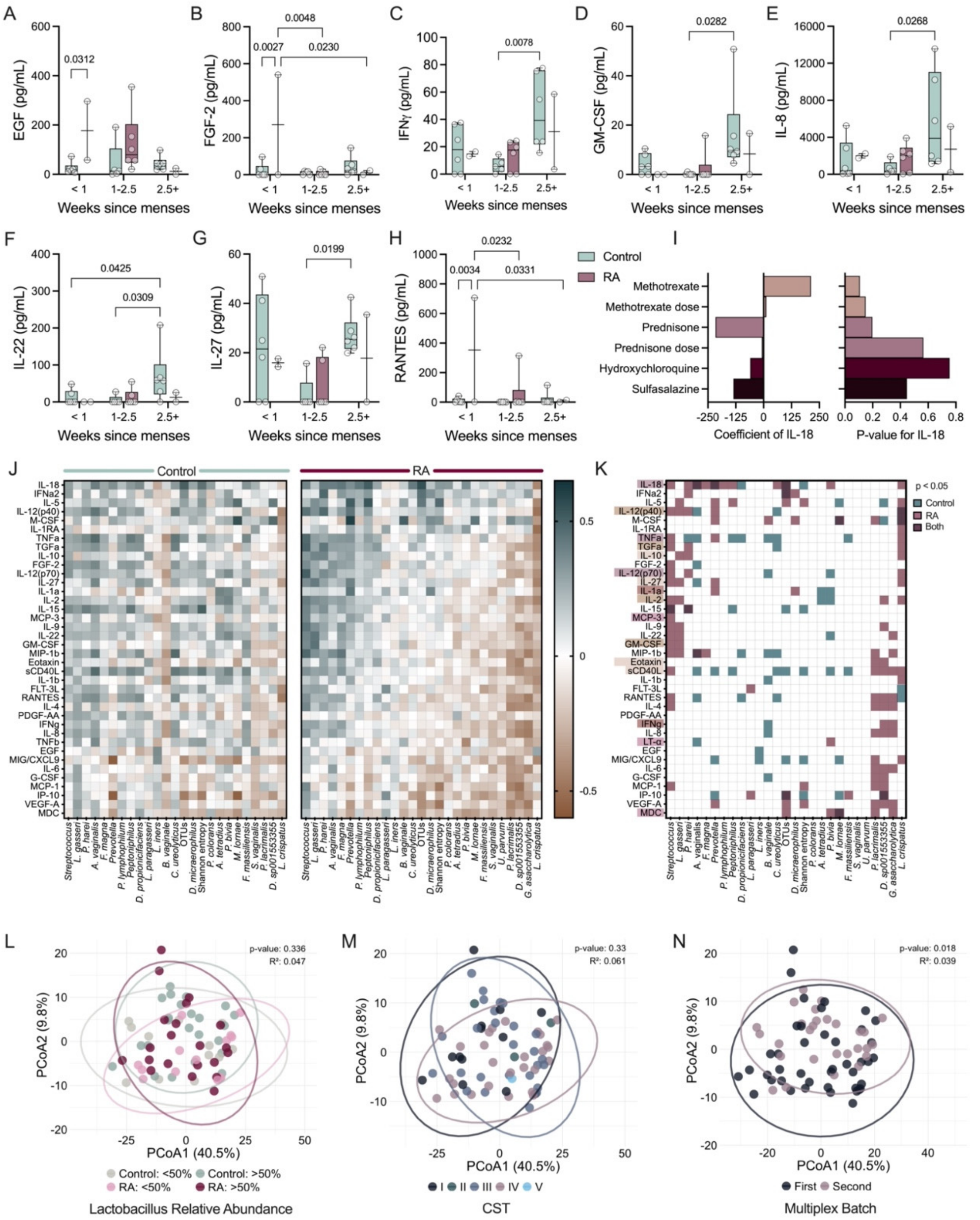

Supplemental Figure 3. Correlations between immune factors and bacteria associated with RA or RA-related phenotypes. Immunomodulatory factor (**A**) EGF, (**B**) FGF-2, (**C**) IFNγ, (**D**) GM-CSF, (**E**) IL-8, (**F**) IL-22, (**G**) IL-27, and (**H**) RANTES levels across the menstrual cycle. (**I**) Coefficients and *P*-values of IL-18 association with medications. Spearman correlation, by effects of *Prevotella*, *Peptoniphilus*, EGF, and the interaction between sCD40L and diet, controlled for menopausal status and clinic. (**J**) Matrices showing variable correlation coefficients between immune factors and significant microbes in control (left) and RA (right) groups. To reduce noise from rare microbes, taxa represent prevalent species identified in 3 or more samples. (**K**) Significant immune-microbe correlations established in the control (teal), RA (pink), or both (purple) vaginal environments. Immune markers are colored by corresponding RA metric associations shown in **Fig. 4**. Data were statistically analyzed by PERMANOVA (A-C), two-way ANOVA with Tukey's multiple comparisons test (D-K), multiple linear regression (L), and Spearman correlation (J-K). Statistically significant *P*-values are shown.

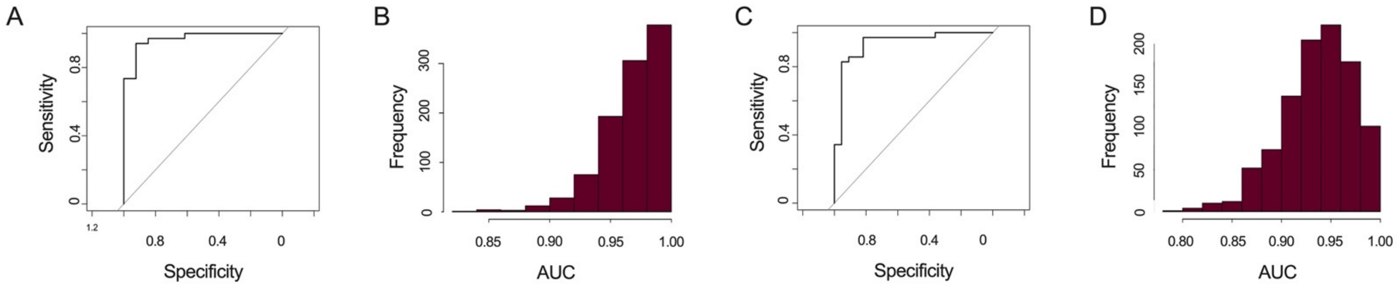

Supplemental Figure 4. Prediction of RA using bootstrapping of a logistic regression model combining diet, microbes, and immune mediators as indicators. *Prevotella*, *Peptoniphilus*, EGF, the interaction between sCD40L and diet, menopausal status, and clinic were incorporated into a logistic regression model predicting RA. (**A**) AUC of 0.97 and (**B**) bootstrap distribution of RA prediction in a cohort of 34 RA and 13 vaginal ACPA negative controls. (**C**) AUC of 0.93 and (**D**) bootstrap distribution of RA prediction using all 35 RA and 22 unique controls (vaginal ACPA positive or unknown). Data were analyzed by multiple logistic regression (A-D). Bootstrap sampling performed with 1000 iterations and seed set at 123 (B,D).

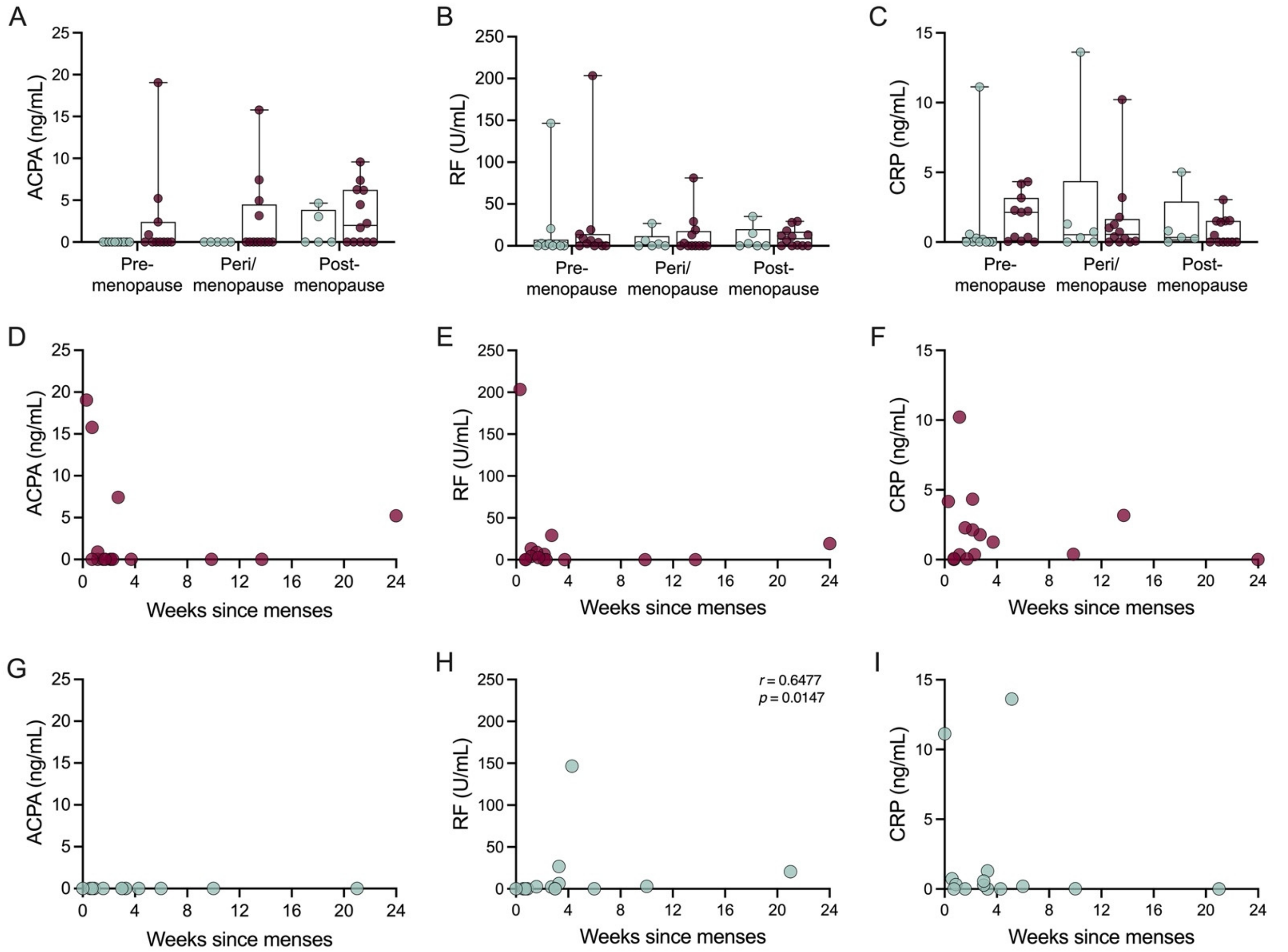

Supplemental Figure 5. RA biomarker levels across menopausal and menstrual stages.
Vaginal concentrations of (**A**) ACPA, (**B**) RF, and (**C**) CRP stratified by menopausal stage in the control and RA groups. Vaginal concentrations of (**D**) ACPA, (**E**) RF, and (**F**) CRP in the RA group and (**G-I**) control group in relation to the start of the most recent menses. Symbols represent individuals. Data in D-I were selected from premenopausal samples depicted in A-C. Data were statistically analyzed by two-way ANOVA with Tukey's multiple comparisons test (A-C) and Spearman correlation (D-I).

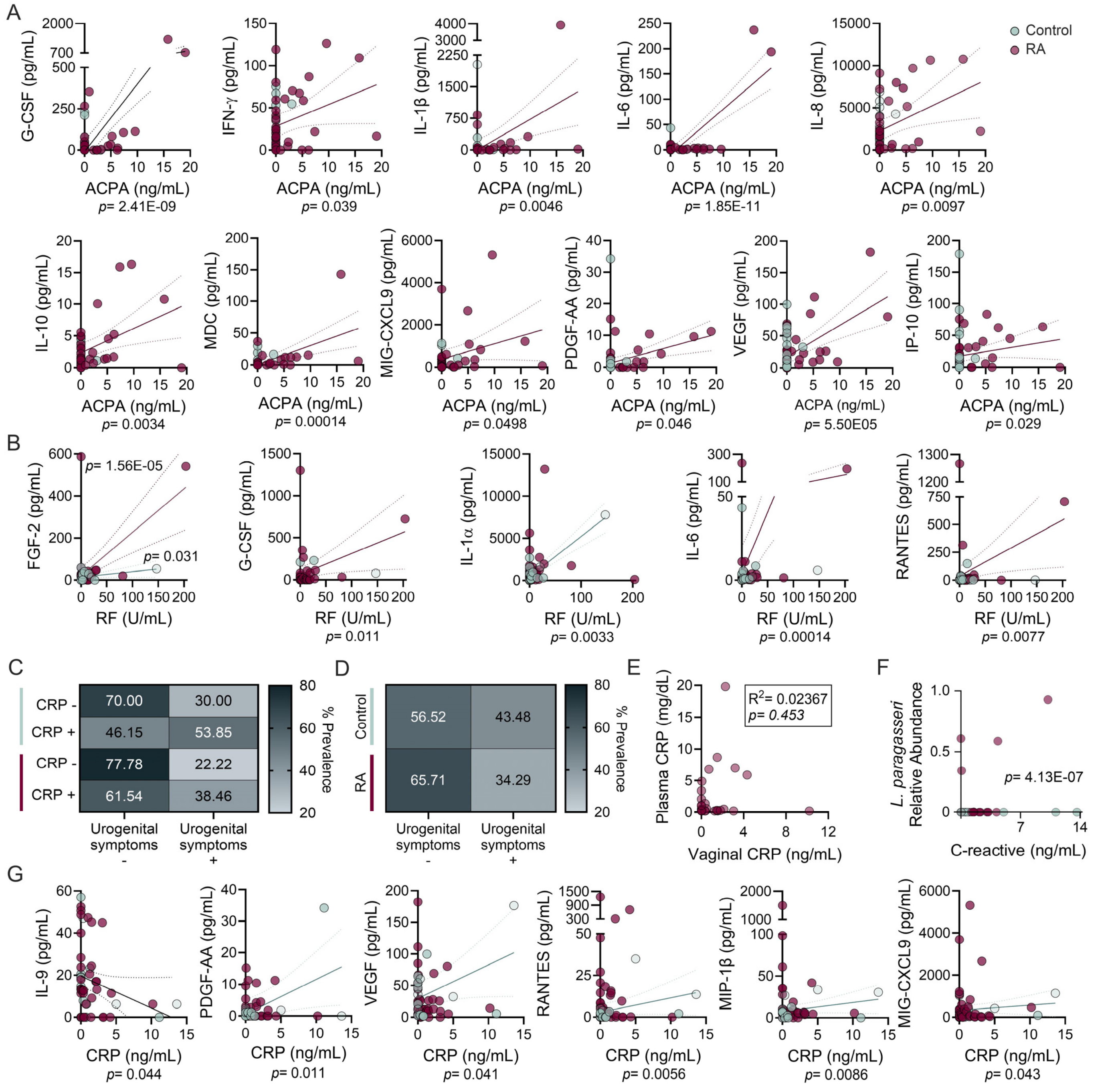

Supplemental Figure 6. Vaginal levels of ACPAs, RF and CRP in correlation with cytokines and clinical history. Correlations between immune factors and vaginal (**A**) ACPA and (**B**) RF. (**C**) Presence of urogenital symptoms according to vaginal CRP+/- status within the control and RA group or (**D**) regardless of CRP detection. (**E**) Correlation between vaginal and plasma concentrations of CRP. Correlations of vaginal CRP and (**F**) *L. paragasseri* or (**G**) immune factors. Data were selected if significant in multiple linear regression analyses (A-B,G) and further assessed by Spearman correlations. Slope and 95% confidence interval are indicated by solid and dashed lines, respectively. Significant correlations in the RA group are dark red and light blue is used for the control group. FGF-2 was significant for each group (B) and IL-9 for both groups were combined (G). Fisher's exact test (A-B) and multiple linear regression (F) were also performed.

Supplementary Table Legends

Supplementary Table 1. Regression models, variables, and statistical parameters. This table contains all regression models used in the manuscript, stratified by individual comparisons and data sets, with additional statistical parameters and significant discoveries indicated in separate columns.

Supplementary Table 2. Clinical demographics represented in multiplex batches. This table contains clinical information of samples represented in each cytokine multiplex run given that cytokine profiles vary across batches.

Supplementary Table 3. Clinical demographics represented in each sequencing batch. This table contains clinical information of samples represented in each of the seven sequencing batches. Variation in the proportion of RA vs control and Clinic demographics were noted.

Supplementary Table 4. Cytokine raw values. This table contains raw values for all cytokines and RA-associated biomarkers at the indicated concentrations in each column.

Table S2. Clinical demographics represented in multiplex batches

**Characteristic**	**Overall** N = 70[1]	**First** N = 42[1]	**Second** N = 28[1]	**p-value**[2]
**RA_status**				0.33
Control	35 (50%)	19 (45%)	16 (57%)	
Rheumatoid Arthritis	35 (50%)	23 (55%)	12 (43%)	
**Age**	46 (21 - 62)	44 (21 - 61)	50 (30 - 62)	0.18
**Age_cat**				0.13
18-30	8 (11%)	7 (17%)	1 (3.6%)	
31-45	26 (37%)	17 (40%)	9 (32%)	
46+	36 (51%)	18 (43%)	18 (64%)	
**Clinic**				0.002
McNair	31 (44%)	25 (60%)	6 (21%)	
Smith	39 (56%)	17 (40%)	22 (79%)	
**Menopausal Status**				0.20
Peri/menopause	22 (31%)	11 26%)	11 (39%)	
Post-menopause	19 (27%)	10 (24%)	9 (32%)	
Pre-menopause	29 (41%)	21 (50%)	8 29%)	
**Diet (Meat/Fat intake)**				0.006
High	48 (69%)	34 (81%)	14 (50%)	
Reduced	22 (31%)	8 (19%)	14 (50%)	
**Recent Period**				0.22
1-2 weeks	12 (17%)	10 (24%)	2 (7.1%)	
month(s)	37 (53%)	19 (45%)	18 (64%)	
Not disclosed	10 (14%)	7 (17%)	3 (11%)	
Within Week	11 (16%)	6 (14%)	5 (18%)	

[1] Median (range) or Frequency (%)

[2] Pearson’s Chi-squared test; Wilcoxon rank sum test; Fisher’s exact test

Table S3. Clinical demographics of samples represented in each sequencing batch

	**Overall** N = 86[1]	**22-Apr** N = 6[1]	**22-Jun** N = 16[1]	**23-Dec** N = 2[1]	**23-Jul** N = 13[1]	**24-Aug** N = 12[1]	**24-Mar** N = 20[1]	**24-May** N = 17[1]	**p-value**[2]
**RA_status**									<0.001
Control	50 (58%)	6 (100%)	16 (100%)	2 (100%)	4 (31%)	7 (58%)	5 (25%)	10 (59%)	
RA	36 (42%)	0 (0%)	0 (0%)	0 (0%)	9 (69%)	5 (42%)	15 (75%)	7 (41%)	
**Age**	47 (21 - 62)	41 (39 - 45)	41 (23 - 62)	45 (29 - 61)	47 (21 - 61)	52 (32 - 62)	47 (27 - 60)	49 (39 - 60)	0.37
**Clinic**									<0.001
McNair	46 (53%)	6 (100%)	16 (100%)	2 (100%)	13 (100%)	1 (8.3%)	8 (40%)	0 (0%)	
Smith	40 (47%)	0 (0%)	0 (0%)	0 (0%)	0 (0%)	11 (92%)	12 (60%)	17 (100%)	
**Menopausal Status**									
Peri/ menopause	25 (29%)	0 (0%)	4 (25%)	0 (0%)	3 (23%)	3 (25%)	4 (20%)	11 (65%)	
Post-menopause	26 (30%)	0 (0%)	5 (31%)	1 (50%)	4 (31%)	5 (42%)	7 (35%)	4 (24%)	
Pre-menopause	35 (41%)	6 (100%)	7 (44%)	1 (50%)	6 (46%)	4 (33%)	9 (45%)	2 (12%)	
**Diet (Meat/Fat intake)**									
High	57 (66%)	6 (100%)	11 (69%)	0 (0%)	12 (92%)	6 (50%)	14 (70%)	8 (47%)	
Reduced	24 (28%)	0 (0%)	4 (25%)	1 (50%)	1 (7.7%)	5 (42%)	5 (25%)	8 (47%)	
Unknown	5 (5.8%)	0 (0%)	1 (6.3%)	1 (50%)	0 (0%)	1 (8.3%)	1 (5.0%)	1 (5.9%)	
**Recent Period**									
1-2 weeks	15 (17%)	1 (17%)	3 (19%)	0 (0%)	2 (15%)	0 (0%)	7 (35%)	2 (12%)	
Month(s)	43 (50%)	2 (33%)	7 (44%)	0 (0%)	6 (46%)	9 (75%)	9 (45%)	10 (59%)	
Not disclosed	16 (19%)	0 (0%)	5 (31%)	1 (50%)	4 (31%)	1 (8.3%)	4 (20%)	1 (5.9%)	
Within Week	12 (14%)	3 (50%)	1 (6.3%)	1 (50%)	1 (7.7%)	2 (17%)	0 (0%)	4 (24%)	

[1] Median (range) or Frequency (%)

[2] Fisher's exact test; Kruskal-Wallis rank sum test; NA
